# INTERSECTING IDENTITIES AND HEALTH-RELATED QUALITY OF LIFE AMONG LGBTQ+ OLDER ADULTS

**DOI:** 10.1093/geroni/igae098.2193

**Published:** 2024-12-31

**Authors:** Hyun-Jun Kim, Hailey Jung, Austin Oswald, Karen Fredriksen-Goldsen

**Affiliations:** University of Washington, Seattle, Washington, United States; University of Washington, Seattle, Washington, United States; Dalhousie University, Nova Scotia, Canada; University of Washington, Seattle, Washington, United States

## Abstract

LGBTQ+ older adults remain understudied in terms of the intersectionality of their identities and its impact on health. It is unclear how certain aspects of identity are perceived as central and how the intersection of multiple identities influences experiences of discrimination. This study identifies intersectional profiles of identity centrality and attributions for day-to-day discrimination, focusing on gender, sexual and gender identity, age, and race/ethnicity among LGBTQ+ older adults. It also examines the associations between profile membership, key psychological and social factors, and physical and mental health-related quality of life (HRQOL). Utilizing data from the National Health, Aging, and Sexuality/Gender Study, the first national longitudinal study of 2,450 LGBTQ+ older adults, we unveiled five latent profiles using Latent Class Analysis, which depict heterogeneous patterns in individuals’ perception of their identities in terms of centrality and as the basis of their discrimination experiences. The most prevalent was the moderate centrality and discrimination profile (C1; 31%), followed by high centrality and discrimination (C2; 26%), low centrality and discrimination (C3; 17%), high centrality and low discrimination (C4; 15%), and low centrality and high discrimination (C5; 11%). C2 and C5 exhibited lower physical and mental HRQOL compared to other profiles, suggesting that multiple discrimination attributes may contribute to compromised health, regardless of identity centrality. C2 and C4 showed high identity centrality across all identities, which was linked to greater critical awareness and activism. This study highlights the significance of incorporating an intersectional lens to better understand the distinct needs and strengths within LGBTQ+ aging communities.

